# The complex transcriptional regulation of heat stress response in maize

**DOI:** 10.1007/s44154-024-00165-x

**Published:** 2024-04-26

**Authors:** Mingxiu Ruan, Heng Zhao, Yujing Wen, Hao Chen, Feng He, Xingbo Hou, Xiaoqin Song, Haiyang Jiang, Yong-Ling Ruan, Leiming Wu

**Affiliations:** 1 The National Engineering Laboratory of Crop Resistance Breeding, School of Life Sciences, Anhui Agricultural University, Hefei, 230036 China; 2 State Key Laboratory of Crop Stress Biology in Arid Areas and College of Horticulture, Northwest A&F University, Yangling, 712100 China; 3 School of Agronomy, Anhui Agricultural University, Hefei, 230036 China; 4 Division of Plant Sciences, Research School of Biology, The Australian National University, Canberra, ACT 2601 Australia

**Keywords:** Heat stress, Maize, Transcriptional regulation, Epigenetic regulation, Interaction

## Abstract

As one of the most important food and feed crops worldwide, maize suffers much more tremendous damages under heat stress compared to other plants, which seriously inhibits plant growth and reduces productivity. To mitigate the heat-induced damages and adapt to high temperature environment, plants have evolved a series of molecular mechanisms to sense, respond and adapt high temperatures and heat stress. In this review, we summarized recent advances in molecular regulations underlying high temperature sensing, heat stress response and memory in maize, especially focusing on several important pathways and signals in high temperature sensing, and the complex transcriptional regulation of *ZmHSFs* (Heat Shock Factors) in heat stress response. In addition, we highlighted interactions between *ZmHSFs* and several epigenetic regulation factors in coordinately regulating heat stress response and memory. Finally, we laid out strategies to systematically elucidate the regulatory network of maize heat stress response, and discussed approaches for breeding future heat-tolerance maize.

## Introduction

As a consequence of global warming, high temperature (HT) weather occurred more frequently than ever before with 2022 being the hottest year in record, which resulted in widespread threats to food security and agricultural sustainability (Challinor et al. 2014; Kan et al. 2023). HT could affect plant growth, development, geographical distribution, as well as crop quality and productivity (Casal and Balasubramanian 2019). In Arabidopsis, mild and moderate ambient temperatures such as 24-30 ^◦^C could accelerate shoot and root growth as well as the transition to flowering and other morphological changes, collectively referred to as thermomorphogenesis (Chen et al. 2022) . However, when on exposure to HT above a threshold level such as 30-37 ^◦^C, Arabidopsis plants typically experience heat stress (HS), impairing respiration, photosynthesis, water and nutrient uptake, and fertility (Djanaguiraman et al. 2020). Indeed, compared to the growth of un-domesticated plant species such as Arabidopsis, cereal crops are highly vulnerable to HS (Challinor et al. 2014). Every 1^◦^C increase in global mean temperature is estimated to reduce the global yield of wheat by 6.0%, rice by 3.2%, maize by 7.4%, and soybean by 3.1% (Zhao et al. 2017; Kraus et al. 2022). As a consequence of significant crop yield losses, it is imperative to develop crops that are capable of adaption and tolerance to HT.

As one of the most important food and feed crops worldwide, maize suffers tremendous damages under HS stress at different growth stages (Djalovic et al. 2024). At seeding stage, temperature over 30 ^◦^C will lead to disrupted water relation, restricted root growth and significant decrease photosynthesis rate in maize (El-Sappah et al. 2022). In reproductive stage, temperature over 35 ^◦^C shortens the reproductive period, decreases pollen fertility and silk elongation, thereby reducing the number of flowers and fruits, and ultimately maize yields (Sánchez et al. 2014). During pollination and grain set, temperatures over 35^◦^C suppress fertilization in maize and decreases its yield by 101 kg/ha per day (Dawood et al. 2020). In China's Huang-Huai-Hai and other dominant maize producing areas, HS have become a frequent natural disaster affecting the safety of maize production (Lobell et al. 2011; Hu et al. 2023). Clearly, it is of major significance to dissect the molecular basis of HS responses to identify candidate genes for heat-tolerant breeding in maize.

Although the molecular mechanisms of plant response to HT have been progressively established and reviewed in model plant Arabidopsis (Ohama et al. 2017; Ding et al. 2020; Chen et al. 2022; Guihur et al. 2022), recent studies have indicated conservative and differential regulations of HS response in major crop plants such as rice (Li et al. 2023a), wheat (Sun et al. 2021) and maize (El-Sappah et al. 2022; Djalovic et al. 2024). In this review, we summarized advances in molecular regulations underlying HT sensing and HS response in maize as well as other cereals where relevant, focusing on the complex transcriptional regulation of *ZmHSFs* in heat stress response. In addition, we highlighted interactions between *ZmHSFs* and several epigenetic regulation factors in heat stress response and memory. Finally, we laid out strategies to systematically dissect the regulatory network of maize HS response, and discussed effective approaches for future heat-tolerance maize breeding.

## Pathways and signals in high temperature sensing

### Pectin in the cell wall

**Fig. 1:**
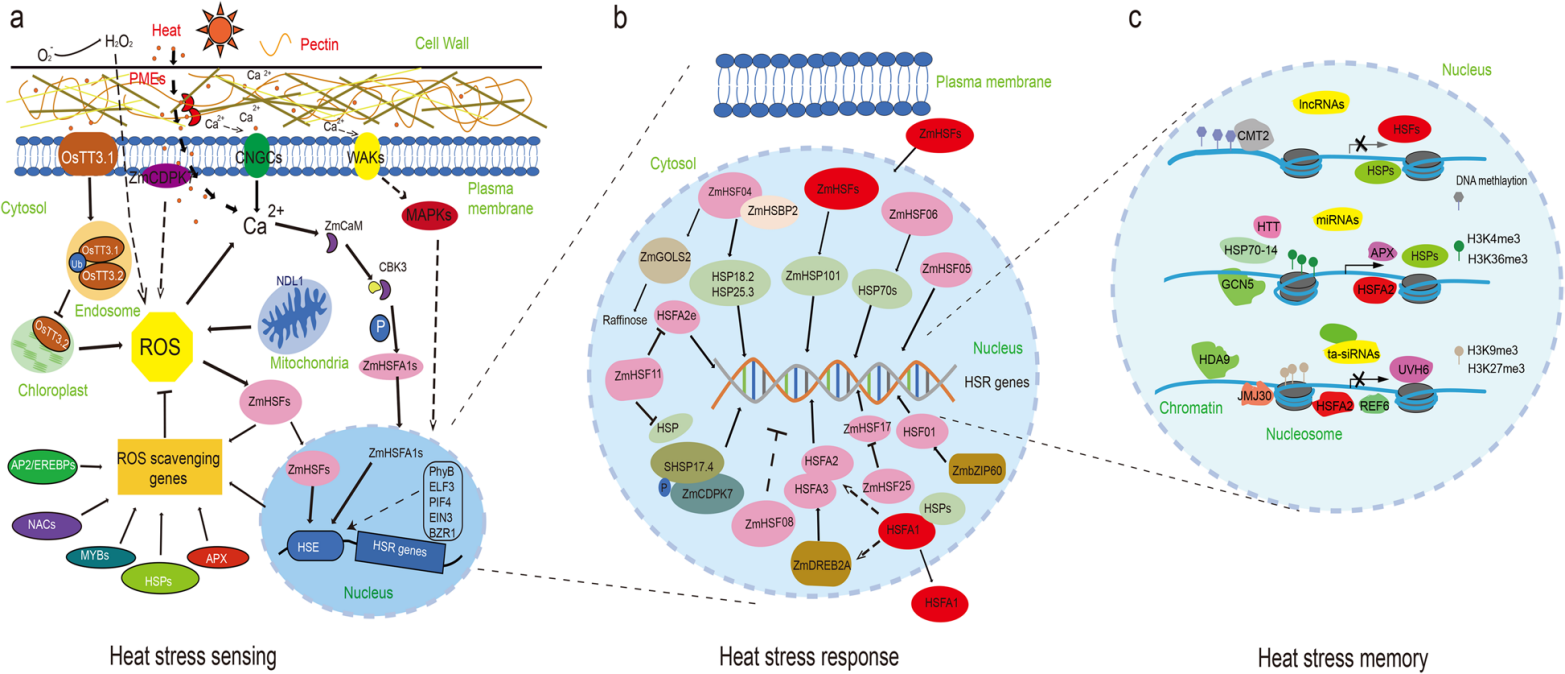
The complex molecular regulations of heat stress response in plants. **a** The major pathways and signals in high temperature sensing including pectin from cell wall, Ca^2+^ signaling channels and sensors in plasma membrane, reactive oxygen species and other nuclear proteins. **b** The transcriptional regulation of heat stress response mainly controlled by HSF-HSP pathway in maize. Several *ZmHSFs* have been identified positively in regulating HS response in plants including *ZmHSF01*, *ZmHSF04*, *ZmHSF05*, *ZmHSF06* and *ZmHSF17*, while *ZmHSF08*, *ZmHSF11*and *ZmHSF25* negatively regulate HS response. Other proteins such as ZmDREB2A and ZmbZIP60 also play functions in heat stress response. Several HSFs could coordinately regulate HS response through protein interaction such as HSFA2 and HSFA3. **c** The epigenetic regulations in heat stress memory. Several DNA methyltransferases such as CMT2, is considered an inhibitory mark in gene expression silencing of HSFs and HSPs. Histone H3 methylated on K4 (H3K4me) and H3K36me mainly generates an open chromatin configuration related to transcription activation. Several genes such as the histone acetyltransferase GCN5 (general control non-depressible 5) promote open chromatins and enhance gene expression. H3K9me3 and H3K27me3 are responsible for closed chromatin states that result in transcriptional repression. Several genes such as JUMONJI (JMJ) proteins, HISTONE DEACETYLASE9 (HDA9) could maintain repressive histone marks on memory genes and repress gene expression of *HSFs*. Several non-coding RNAs trans-acting small interfering RNAs (ta-siRNAs), microRNAs (miRNAs), and long non-coding circular RNAs (lncRNAs) also play an indispensable role in regulating heat stress response

Plants have evolved a series of molecular pathways in HT sensing and heat-induced signal cascades (El-Sappah et al. 2022). Multiple cellular and subcellular components can sense HT and subsequently activate an arrow of signaling cascades for rapid adaptive modification (Fig. 1a). For example, the cell wall is the first protective barrier in plant cells, and senses HT via a complex signal transduction (Wu et al. 2018; Wolf 2022). Moderate HS could activate PME (pectin methyl-esterase) enzyme activity in de-esterification of pectin which caused cell wall loosening and Ca^2+^ mobilization from apoplast to the cytoplasm (Wu et al. 2018; Wan et al. 2021). The oligogalacturonic acids derived from pectin could be further perceived by WAKs (wall-associated kinase) extracellular domain to activate MAPK (mitogen-activated protein kinases) cascade for HS sensing and response (Decreux and Messiaen 2005; Kohorn et al. 2009; Brutus et al. 2010). Recently, the expression of *GhCYP703A2* and *GhQRT3*, two polygalacturonases were found to be inhibited under HT by affecting pectin metabolism and sporopollenin synthesis, and resulting in abnormal pollen wall development and male sterility in cotton (Li et al. 2023c). The gene expression of a wall-associated RLK-like (WAKL) gene *CaWAKL20* from pepper (*Capsicum annuum L.*) was inhibited by HS, and silencing of *CaWAKL20* could enhance pepper thermotolerance (Wang et al. 2019). Therefore, alterations of the component and modification of pectin could affect plant HT sensing by remodeling cell wall structure, and the molecular regulation of pectin synthesis and other related genes such as *ZmWAKs* need to be further studied in maize.

#### Thermosensors at plasma membrane

Increasing evidence proves the primary HT sensing occurs at the plasma membrane through many thermosensors in plants (Bourgine and Guihur 2021; Mittler et al. 2011). Any protein could be defined as a thermosensor not only able to perceive high temperature increments but also trigger specific signaling pathways which can upregulate related heat stress response (HSR) genes (Vu et al. 2019; Mittler et al. 2011). For example, Cyclic Nucleotide-Gated Channels (CNGCs) act as thermosensors embedded in the plasma membrane to allow the inward flux of calcium and activate the HS response (Finka et al. 2012). There are 11 plasma membrane-localized cyclic nucleotide-gated ion channels (*CNGC)* genes identified to be Ca^2+^ conducting channels of maize in heat sensing (Hao and Qiao 2018). In response to heat, Ca^2+^ acts as an important messenger that binds directly to CaM (Calmodulin) and change their conformation to elicit HSR (Dodd et al. 2010). CaM-binding protein kinase CBK3 is a positive regulator in the HS signal transduction by phosphorylating *HsfA1* and enhancing its DNA-binding activity (Liu et al. 2018b). ZmCDPK7 is another plasma membrane-anchored protein under normal conditions, but could be also localized to the cytoplasm under HS to induce Reactive Oxygen Species (ROS) accumulation and enhance heat-stress tolerance by phosphorylating sHSP17.4 and respiratory burst oxidase homolog RBOHB in maize (Zhao et al. 2021). Recently, a new sensor from plasma membrane, *Thermo-tolerance 3* (*TT3*) , consisting of two genes, plasma membrane–localized E3 ligase *TT3.1* and chloroplast precursor protein *TT3.2*, could interact together in endosome to promote the degradation of *TT3.2* to protect chloroplast from heat stress damage and reduce grain-yield losses under heat stress in rice (Zhang et al. 2022; Li and Liu 2022a). In future studies, it would be exciting to identify more sensors of HS located in plasma membrane in major crop plants, such as maize.

#### Reactive oxygen species as signal molecules

ROS is an important signaling molecule that could stimulate or interact with the Ca^2+^ signaling pathways during heat stress sensing (Li et al. 2018). HS alters the normal status of the chloroplast and mitochondria membranes, causing the over-accumulation of ROS (Navarro et al. 2021). For example, *NDL1* encodes a mitochondria localized ATP-dependent metalloprotease essential for regulating thermotolerant maize growth by altering endogenous auxin levels, and *needle1* (*ndl1*) is a temperature-sensitive mutant with hyper-accumulate ROS owing to respiratory defects in mitochondria (Liu et al. 2019a). When the accumulated ROS elicit cell damage, antioxidants and ROS scavenging genes are activated to maintain cellular redox homeostasis and HS acclimation (Choudhury et al. 2017). In response to ROS, HSR genes, such as several *MYBs*, *AP2*/*EREBPs*, *NACs*, *HSPs*, *Rubisco* and *APX* and are activated to scavenge the accumulated ROS (Jagtap et al. 2020; Khan et al. 2023). ROS may activate *HSFA1* by inducing their trimerization, which, in turn, enhanced their DNA binding activity with the targeting HSR genes (Liu et al. 2013). Therefore, it is important to explore and identify the genes which generate or eliminate ROS for HT sensing and responsing in maize.

#### Other proteins

Other proteins are also participated in thermosensing. Phytochromes function in thermosensing and signal transduction pathways during heat stress (Jung et al. 2016). PhyB (Phytochrome B) signaling is exacerbated by warm temperature during early night in Arabidopsis (Vu et al. 2019). The basic-helix-loop-helix transcription factors PIF4 (PHYTOCHROME-INTERACTING FACTOR 4) and PIF7 are two master regulators in thermomorphogenesis (Koini et al. 2009; Kumar et al. 2012). PIF4, is involved in promoting auxin-dependent growth in warm conditions (Zhao and Bao 2021). The protein level of PIF7 is enhanced by conformational changes in its RNA secondary structures in response to higher temperatures in Arabidopsis (Chung et al. 2020). ELF3 (EARLY FLOWERING 3) containing a prion-like domain functions as a repressor and negative regulator of thermomorphogenesis in Arabidopsis (Jung et al. 2020). Moreover, several hormones have multiple functions in the response to HS. EIN3 (ETHYLENE-INSENSITIVE 3) is a key transcriptional regulator of the ethylene signaling pathway and higher temperatures promote EIN3 protein accumulation (Hao et al. 2021). BRASSINAZOLE RESISTANT1 (BZR1) is a positive regulator of BR (Brassinosteroid) signaling, and over-expression of BZR1, improves thermotolerance by enhancing H_2_O_2_ levels in tomato (Yin et al. 2018). In maize, these related proteins are less studied to regulate HT sensing. In the future, more genes related to other traits of HS sensing could be identified in different maize populations by quantitative trait locus (QTLs) mapping and genome-wide association study (GWAS). For example, four QTLs and 17 genes associated with 42 single nucleotide polymorphisms (SNPs) related to thermotolerance of seed-set had been identified by mapping of QTLs and GWAS of 261 diverse maize lines (Gao et al. 2019).

## The transcription regulation of HS response

### HSFs act as master transcription factors in HS response

After plants sense HS signals, a primary response occurs mainly by heat-induced proteins such as heat shock transcription factors (HSFs) to active downstream heat shock proteins (HSPs) and other HSR gene expression to mitigate the effect of HS, which is conserved across different species (El-Sappah et al. 2022; Guihur et al. 2022). Plants have evolved multiple HSFs to participate in HS response, which are divided into three classes: HSFA, HSFB, and HSFC according to their structural characteristics and phylogenetic comparisons (Lin et al. 2011; Zhang et al. 2020a). Class A HSFs contribute to transcriptional activation, whereas the rest two classes have no specific role in transcriptional activation and might be serving as coactivators cooperating with class A (Haider et al. 2022). HSFA1 is the master transcription activator inducing expression of different HSFs by binding the HS elements in their promoter regions, including dehydration responsive element binding protein2A (DREB2A), ERF/AP2, HSFA2, HSFA7 and HSFBs, which has been proved to be conservative in different plant species (Yoshida et al. 2011). HSFA1 activates HSFA2 and prolongs the acquired thermotolerance by maintaining HSP expression for HS generational memory (acquired thermotolerance for several days) in Arabidopsis (Charng et al. 2023). HSFA1 could interact with HSP70 and HSP90 to repress HSFA1 activity under normal conditions in tomato (Hahn et al. 2011). HSFA1 proteins could also regulate self-activity by post-translational modification, phosphorylation and SUMOylation in Arabidopsis and wheat (Rytz et al. 2018; Ding et al. 2020; Wang et al. 2023). HSFA1s are required for PIF4 accumulation at a warm daytime temperature through protein interaction, and play a critical regulator in integrating both thermomorphogenesis and HS responses in Arabidopsis (Li et al. 2024a; Tan et al. 2023 Science Advance). HSFA1 was also regulated by miR165 and miR166 and their target transcript, PHABULOSA (PHB), at the transcriptional and translational levels to control plant HS responses in Arabidopsis (Li et al. 2023b). What is more, the HS-induced epigenetic states, can be transmitted to the next generation (transgenerational memory which is another type of HS memory) through meiosis (Liu et al. 2019b; Zhu et al. 2023). Therefore, the HSF gene family plays an important and complex transcription regulation on downstream HSPs and HSR gene expression in HS response and memory.

### Identification and function study of ZmHSFs

In maize, there are 31non-redundant HSFs and might have multiple regulations on different stress responses (Zhang et al. 2020a). We summarized recent functional studies of *ZmHSFs* as shown in Fig. 1b and Table 1. *ZmHSF01*, *ZmHSF03*, and *ZmHSF23* was observed with higher expression exposed to heat stress probably proving their significant roles in regulating heat stress response (Lin et al. 2011). In Arabidopsis seedlings, *ZmHSF01* (*ZmHSFTF13*) compensated for the thermotolerance defects of mutant *athsfa2*, and *ZmHSF01*-overexpressing lines showed enhanced basal and acquired thermotolerance (Zhang et al. 2020b). ZmHSFA2 (ZmHSF04) and HEAT SHOCK BINDING PROTEIN 2 (ZmHSBP2) physically interact with each other and antagonistically modulate expression of *GALACTINOL SYNTHASE2* (*ZmGOLS2*) and raffinose biosynthesis in transformed maize protoplasts and Arabidopsis plants (Gu et al. 2019). Ectopic overexpression of *ZmHSF04* confers increased thermal and salt‑stress tolerance in transgenic Arabidopsis by up-regulated the expression of heat-specific *HSP* genes (*AtHsp25.3*, *AtHsp18.2*, and *AtHsp70B*) and stress-related genes (*AtAPX2* and *AtGolS1*) compared to the wild type (Jiang et al. 2017). *ZmHSF05* improves thermotolerance by up-regulating *Hsps* in *Arabidopsis thaliana* and rescues thermotolerance defects of the *athsfa2* mutant (Li et al. 2019b). *ZmHSF06* is the specific gene localized in pollens and regulate *Hsp70-2* and *Hsp70-4* in response to HS (Li et al. 2015). Overexpression of *ZmHSF06* in Arabidopsis plants have enhanced basal and acquired thermotolerance, stronger drought-stress tolerance and growth advantages under mild heat stress conditions (Li et al. 2015). Overexpressing of *ZmHSF17-II*, an intron retention isoform of subclass A2 gene *ZmHSF17*, could increase the thermotolerance in Arabidopsis (Zhang et al. 2024). *ZmHSF08* from class B HSFs was induced by salt, drought, and abscisic acid (ABA) treatment, and negatively regulates many stress/ABA response genes under salt stress and drought stress (Wang et al. 2021). *ZmHSF11*, also a member of class B HSFs, decreases plant tolerance to heat stress by negatively regulating the expression of oxidative stress-related genes *HSP17* and *HSFA2e* (Qin et al. 2022). Recently, *ZmHSF20* of class B2a was reported as a negative factor in regulating heat stress through the ZmHSF20-ZmHSF4-ZmCESA2 module (Li et al. 2024b). *ZmHSF20* could bind the promoters of *ZmCESA2* and *ZmHSF4*, while *ZmHSF4* directly activated the expression of *ZmCESA2* to positively regulate heat response (Li et al. 2024b). Although many *ZmHSFs* have been cloned and studied in Arabidopsis, the real regulatory functions of *ZmHSFs* in response to different stress need to be validated in maize by creating overexpressing and knockout transgenic plants in maize. Furthermore, the down-regulated and interaction network of *ZmHSFs* in responses to HS need to be systematically studied to extend our understanding of the complexity of the HS regulation in maize beyond Arabidopsis.

**Table 1:** Reported transcription factors involved in the regulation of heat stress response in maize

**Gene**	**Functions in response to stresses**	**References**
ZmHSF01	ZmHsf01 positively regulate thermotolerance probably by H3K9 hyperacetylation in the promoter region	Zhang et al. 2020b; Lin et al. 2011
ZmHSF03	Heat stress response	Lin et al. 2011
ZmHSF04	ZmHsf04-overexpressing increased thermal and salt-stress tolerance	Lin et al. 2011; Jiang et al. 2017; Zhang et al. 2020a
ZmHSF05	ZmHsf05 could improve drought tolerance and thermotolerance	Li et al. 2019; Zhang et al. 2020a
ZmHSF06	ZmHsf06 could enhance thermal and drought-stress tolerance	Li et al. 2015
ZmHSF08	ZmHsf08 negatively regulates many stress/ABA response genes under salt stress and drought stress	Wang et al. 2021
ZmHSF11	ZmHsf11 decreases plant tolerance to heat stress	Qin et al. 2022; Lin et al. 2011
ZmHSF17	Heat stress response	Lin et al. 2011; Zhang et al. 2024; Li et al. 2024b
ZmHSF23	Heat stress response	Lin et al. 2011
ZmHSF25	Heat stress response	Lin et al. 2011; Li et al. 2024b
ZmHSF28	ZmHsf28 improves drought tolerance in the monocot maize and the dicot Arabidopsis	Liu et al. 2023b
ZmDREB2A	ZmDREB2A plays an essential role both in heat and drought tolerance in maize	Qin et al. 2007
ZmMYB-R	ZmMYB-R was induced when maize exposed to abiotic stress factors including heat, drought and cold	Kimotho et al. 2019
ZmbZIP60	ZmbZIP60 activates the expression of ZmHSF01 and upregulates a constellation of HSP genes	Li et al. 2020b

### The interactions between HSFs in coordinately regulating heat stress response

There is increasing evidence for the coordinate regulation of *HSFs* in HS response by protein interaction. The interactions could exist in different isoforms of one HSF, or the same subfamily of HSFs or different subfamily of HSFs. For example, ZmHSF17-I and ZmHSF17-II, two different isoforms of *ZmHSF17* by alternative splicing, could interacted with each other to negatively regulate its own transcription under heat stress (Zhang et al. 2024). HSFA3 could interact with the same sub-family gene HSFA2 to form heteromeric complexes that efficiently promotes transcriptional memory genes such as *HSA32*, *APX2* and *HSP22* by influencing histone H3 lysine4 (H3K4) hyper-methylation following HS exposure in Arabidopsis (Friedrich et al. 2021). Recently, *LIHSF2C*, a class C HSF involved in thermotolerance, could not only interact with itself, but also interact with LlHSFAs of lily, AtHSFA1e and AtHSFA2 of Arabidopsis, and NbHSFA2 of tobacco (Wu et al. 2024). After suffering HS, the homologous interaction of LlHSFC2 was repressed, but the heterologous interaction between LlHSFC2 and HSFAs was promoted, which exerted its co-activation effect for thermotolerance establishment and maintenance (Wu et al. 2024). Even though theses interactions between HSFs have been identified in many plants, the coordinated mechanisms need to be further explored to understand the fine regulation of HS response especially in maize.

### HSPs, not just downstream genes of HSFs in HS response

Heat shock proteins (HSPs) have multiple functions in HS response and regulation. First, HSPs including HSP20, HSP60, HSP70, HSP90, HSP100, and HSP110 are induced by *HSFs*, and act as chaperones to renature misfolded proteins that are important for thermotolerance in Arabidopsis and other plants (Lee et al. 2007; Kotak et al. 2007; EI-Sappah et al. 2022). However, single mutants of these HSPs do not cause severe HS-deficient phenotype indicating the functional redundance of HSPs in HS response (Guihur et al. 2022). HSP101 is one of key HSPs for the acquisition of thermotolerance in different plants (Gurley 2000). In rice, *OsHSP101* acts as positive regulator of thermotolerance and heat memory in rice (Lin et al. 2014). Transcriptome data showed that *ZmHSP101* is highly expressed in male meiocytes under normal growth conditions (Dukowic-Schulze and Chen 2014). Recently, *ZmHSP101* was found to function in DNA double-strand breaks repair and subsequent meiosis. Overexpression of *ZmHSP101* in anthers results in robust microspores with enhanced heat tolerance (Li et al. 2022c). In addition, abscisic acid-induced calcium-dependent protein kinase ZmCDPK7 interacts with the small heat shock protein sHSP17.4 through phosphorylation, and participates in thermotolerance in maize (Zhao et al. 2021).

In addition, multiple isoforms of HSP and HSF could form complexes and function downstream in determining transcriptional levels. For example, to prevent HSFA1 proteins from being activated under normal temperature conditions, HSP70 forms a complex with HSFA1s, repressing their DNA binding activity and nuclear localization of HSFA1 under permissive temperatures, while HS activates *HSFA1* by loosening the complex in tomato (Yamada et al. 2007; Hahn et al. 2011; Ding et al. 2020). Although HSPs are crucial in the regulation of HS, the specific functions with HSFs and targets of HSPs in major crop plants are largely not yet clear.

### Other transcription factors in regulating heat stress response

Beside HSF family genes, some other different transcription factors also play functions in HS response in plants. A lily membrane-associated NAC transcription factor LlNAC014 increased thermotolerance by activating the DREB2-HSFA3 module in lily (*Lilium longiflorum*) (Wu et al. 2023). DREB2A (DEHYDRATION-RESPONSIVE ELEMENT-BINDING2A) could regulate the expression of *HSFA3* and downstream HSR genes by forming a trimer complex comprised of NUCLEAR FACTOR Y, SUBUNIT A2 (NF-YA2), DNA POLYMERASE II SUBUNIT B3-1(DPB3-1), and NF-YB3 in Arabidopsis (Schramm et al. 2008; Ohama et al. 2017; Ding et al. 2020). BRI1-EMS-SUPPRESSOR 1 (BES1), which is a key regulator of brassinosteroids (BRs) signalling, could be de-phosphorylated mediated by ABA-controlled PP2C phosphatases and activated even in the absence of BRs by heat (Yao et al. 2022). Furthermore, the activated BES1 could interact with HSFA1a and enhance its binding activity to targeted HSEs whether or not under the regulation of ABA or BR (Albertos et al. 2022). REVEILLE4 (RVE4) and REVEILLE8 (RVE8) are two transcription factors of Arabidopsis involved in regulating circadian clock and early HS-induced gene expression, indicating that plants can redeploy and coordinate multiple regulatory networks to adapt to the changing environment (Li et al. 2019a). Heat stress *bzip28* and *bzip60* double-mutant plants are sensitive to heat stress, and ZmbZIP60 activates the expression of a type-A HSFTF13, which, in turn, upregulates the expression of a constellation of *HSP* genes (Li et al. 2020b). ZmDREB2A plays an essential role both in both heat and drought tolerance in maize (Qin et al. 2007). These results indicate heat stress is connected with other abiotic stresses, and plants have evolved other compensating pathways except HSFs to adapt and combat heat stress.

## The interactions between HSFs and epigenetic factors in regulating heat stress memory

### Heat stress memory

HS memory is an important and active process that allow plants to acquire thermotolerance to respond more efficiently when plants encounter stresses more than once. Plants have evolved several HS memory strategies, including generational memory (over several days or weeks) (Perrella et al. 2022; Balazadeh 2022) and transgenerational memory (transmitted to the next generation) (Gallusci et al. 2023). Epigenetic regulation of the HS mainly includes DNA methylation, histone modification and modulation by small non-coding RNA. These epigenetic modifications can activate or repress transcription by generating either 'open' or 'closed' chromatin configurations to regulate the accessibility of HSF transcriptional regulators to HSR genes (Ueda and Seki 2020). We evaluated recent epigenetic modifications of HSF and HSR genes in regulating HS memory as shown in Fig. 1c and Table 2.

**Table 2:** Researches on epigenetic modifications in response to heat stress in plants

**Epigenetic modifications**	**Genes**	**Description**	**References**
DNA and Histone methlaytion	CMT2 (chromomethylase 2)	CMT2-associated CHH methylation levels negatively correlated with thermotolerance	Shen et al. 2014
	H3K4me, H3K36me	generate an open chromatin configuration related to transcription activation	Liu et al. 2010; Ueda and Seki 2020
	H3K9me, H3K27me	generate closed chromatin states that result in transcriptional repression	Liu et al. 2010; Ueda and Seki 2020
	H3K4me2, H3K4me3	provide higher and longer expression of HS memory genes for remembering acquired thermotolerance	Liu et al. 2018a; Shekhawat et al. 2021
	JMJ (jumonji)	regulate H3K27me3, and could maintain repressive histone marks on memory genes	Song et al. 2021; Yamaguchi et al. 2021; He et al. 2021
Histone acetylation	GCN5 (general control non-depressible 5)	enhance histone H3 acetylation and lead to higher transcription of HSFA2, HSFA3, and UVH6 in Arabidopsis	Hu et al. 2015
	HD2C (histone deacetylases 2C)	acts as a transcriptional repressor of heat-activated genes by removing lysine acetylation	Buszewicz et al. 2016
	HDACs (histone deacetylases)	HDA9 and HDA19 are positive regulators while HDA15 is a negative factor in response to elevated temperatures	Shen et al. 2019
	HDA9	heat induced cytoplasm-to-nucleus translocation of HDA9 and promotes the eviction of H2A.Z from nucleosomes of YUC8	van der Woude et al. 2019; Niu et al. 2022
Non-coding RNAs	miRNAs	induced by HS in Arabidopsis, poplar, wheat and maize	Sunkar et al. 2012; Li et al. 2020a; Zhao et al. 2022; Hu et al. 2022
	ta-siRNAs	decrease the abundance of TAS1 under HS leading to higher expression of HSRs	Li et al. 2014
	siRNA	mediate the silencing of ONSEN which could be bound by HSFA2 through heat-response element	Cavrak et al. 2014; Gu et al. 2023
	lncRNAs	respond to stress through various mechanisms	Song et al. 2020; Zhao et al. 2022; Hu et al. 2022
	circRNA	interact with other regulators to control the expression of the HSR genes	He et al. 2020

#### DNA methylation in response to HS

Methylation is the most common form of DNA modification, involving the addition of methyl groups to DNA molecules, thereby altering DNA structure and gene expression, ultimately impacting the plant growth, development, and responses to stressors (Law and Jacobsen 2010; Liu et al. 2023a). 5mC modification catalyzed by DNA methyltransferases, and is considered an inhibitory mark in gene expression, transposon insertion, and excision, as well as genome stability (Kong et al. 2023). HS could induce DNA hypomethylation and hypermethylation. On the other hand, these marked DNA may be carried to the next generation making the progenies ‘primed’ for HS responses (Arora et al. 2022). For example, natural *CMT2* (*Chromomethylase 2*) variation is associated with genome-wide methylation changes, and *cmt2* mutants shew more tolerance to heat-stress, which suggests genetic regulation of DNA epigenetic modifications as a likely mechanism underlying natural adaptation to high temperatures (Shen et al. 2014). However, it still lacks the epigenomic landscape of DNA methylation in response to HS in maize.

#### Histone methylation in regulating HSR genes

Histone methylation is another epigenetic mechanism that plays a key role in mediating plant responses to HS (He et al. 2021). Current researches on histone methylation mainly focus on histones H3 and H4, in which H3K4, K9, K27, and K36 are methylated (He et al. 2021). Histone H3 methylated on K4 (H3K4me) and K36 (H3K36me) mainly generates an open chromatin configuration related to transcription activation, while H3K9me and H3K27me are responsible for closed chromatin states that result in transcriptional repression (Liu et al. 2010; Ueda and Seki 2020). After a treatment of mild primary HS, di- and tri-methylated histone H3 (H3K4me2 and H3K4me3) were enriched at loci of HS memory genes such as *APX2*, *HSP18.2* and *HSP22*, to provide higher and longer expression of these HS memory genes for remembering acquired thermotolerance (Liu et al. 2018a; Shekhawat et al. 2021). JUMONJI (JMJ) proteins such as JMJ30 and JMJ12, are demethylases involved in regulating H3K27me3, and could maintain repressive histone marks on HT memory genes in Arabidopsis (He et al. 2021; Song et al. 2021; Yamaguchi et al. 2021). So, it is interesting to identify genes regulating histone methylation and demethylation in response to HT memory in maize in future studies.

#### Histone acetylation in HS memory

Besides methylation, histone acetylation also plays an important role in plant heat response. Acetylation of histone weakens the interaction of DNA with histone and promotes chromatin decondensation to enhance the transcriptional activity. Histone deacetylation is mainly catalyzed by histone deacetylases (HDACs), which play different roles in response to HS. HD2C acts as a transcriptional repressor of heat-activated genes by removing lysine acetylation at chromatin loci of heat activated genes (Buszewicz et al. 2016). HDA9 (HISTONE DEACETYLASE 9) relocates from the cytosol to the nucleus and positively modulates heat shock signal transduction (Niu et al. 2022). It is also proved that this mechanism of heat-induced cytoplasm-to-nucleus translocation of HDA9 is conserved in wheat and rice, indicating its potential use in crop breeding during global climate warming (Niu et al. 2022). In addition, HDA9 keeps stabilized in response to HT and mediates the histone deacetylation of YUCCA8, an important enzyme in auxin biosynthesis, and promotes the eviction of H2A.Z from nucleosomes resulting in the binding and transcriptional activation of PHYTOCHROME INTERACTING FACTOR 4, revealing the crosstalk between histone modification and chromatin remodeling in HT responses (van der Woude et al. 2019). The functions of these *HAD* genes in maize remain unknown and need to be further studied in regulating HT response and memory.

#### Non-coding RNAs in HS memory

Non-coding RNAs including trans-acting small interfering RNAs (ta-siRNAs), microRNAs (miRNAs), and long non-coding circular RNAs (lncRNAs) play an indispensable role in regulating HS (Liu et al. 2015). Several miRNAs such as miR398, miR156, miR159 and miR160 are found to be induced by HS in Arabidopsis, poplar and wheat (Sunkar et al. 2012). Under HS, the abundance of ta-siRNAs-trans-acting siRNA precursor 1 (TAS1) was decreased leading to higher expression of *HTT* gene expression levels which is cofactors of HEAT SHOCK PROTEIN 70-14 (HSP70-14) to mediate thermotolerance (Li et al. 2014). The OsSGS3-ta-siRNA-OsARF3 module orchestrates trade-offs between thermotolerance and defense in rice (Gu et al. 2023). Other microRNAs such as miR398 and miR156 are positive regulators of some important transcription factors and downstream genes such as *CSDs* (*Copper/zinc Superoxide Dismutase*) and *SPL13* (*Squamosa Promoter-Binding Protein-Like 13*) to maintain thermotolerance (Matthews et al. 2019; Li et al. 2020a). When plants suffer HT damage, lncRNAs can respond to stress through various mechanisms, including the accumulation of osmoprotectants (e.g., proline), calcium ion regulation, stomatal regulation, and hormone signaling and synthesis (Song et al. 2020). Recently, another category of small RNAs called circRNA was found to be involved in HS possibly by interacting with other regulators to control the expression of the HSR genes (He et al. 2020). In maize, by applying a high-throughput sequencing of IncRNAs and sRNA in inbred line CM1, 993 lncRNAs and 340 miRNAs were identified with significantly differential expression under heat stress compared to normal conditions, and constructed a lncRNA-mediated regulatory network to help visualize the molecular response mechanism of IncRNA and mi-RNA in response to heat stress (Zhao et al. 2022; Hu et al. 2022).

#### The coordination between HSFs and epigenetic factors in regulating HS memory

Plants have evolved a complex molecular mechanism in retaining, sustaining and transmitting HS memories, especially by the coordination between HSFs and epigenetic factors (Sharma et al. 2022; Zhu et al. 2023). For example, HSFA2 plays a key role in regulating HS memory by cooperating with histone methylation. HSFA2 is necessary for the sustained accumulation of H3K4 hypermethylation at the promoter region of HS memory genes in response to repeated heat stress (Lämke et al. 2016). This mechanism is only transiently associated with heat stress memory loci in the hours following heat stress. In addition, HSFA2 could also activate H3K27me3 demethylase *RELATIVE OF EARLY FLOWERING 6* (*REF6*), and feedback form a positive loop to transmit transgenerational memory of heat by derepressing HSFA2 (Liu et al. 2019b). Another example is the histone acetyltransferase *GCN5* (*GENERAL CONTROL NON-DEPRESSIBLE 5*) enhanced levels of histone H3 acetylation (H3K9/14ac) at the promoter regions of the *HSFA3* and *UV-HYPERSENSITIVE 6* (*UVH6*) genes leading to higher transcription of *HSFA2*, *HSFA3*, and *UVH6* and confers thermotolerance in Arabidopsis (Hu et al. 2015). What is more, siRNAs are also mediate the silencing of a Copia-type retrotransposon named ONSEN which could be bound by HSFA2 through heat-response element (Cavrak et al. 2014). Therefore, HSF-mediated epigenetic regulation may be a widely adopted mechanism in heat stress memory in plants, which need to be studied in maize.

## Conclusions and perspective

### The complex transcriptional regulation network of heat stress response in maize

Since high temperature and heat damage significantly affect plant growth, development, crop yield and quality, especially in maize, it is imperative to understand the molecular mechanisms and regulatory networks involved in the HS response. Plants have evolved a series of molecular mechanisms to sense heat stress accurately and induce different genes and signal cascades (El-Sappah et al. 2022). The transcriptional regulation by heat-induced HSFs is as a primary heat response to activate *HSPs* and other *HSR* genes to mitigate the effect of heat stress. Epigenetic regulation is also pivotal for heat stress responses, especially in heat stress memory. Furthermore, more studies revealed the coordination between different transcription factors and epigenetic factors is essential for the precise regulation for plant temperature responses and phenological adaption (Zhu et al. 2023). Although these mechanisms have been well studied in Arabidopsis, the conservative and differential regulation in other crop plants such as maize need to further explained. In the future, more transcriptional and epigenetic factors related to HS could be further identified by constructing the gene regulatory network and epigenetic landscape of HS, and their complex interactions of HS response need to be systematically explained to decipher the complex transcriptional regulation network of heat stress response in maize.

### Multi-omics help dissect the complex regulatory network in heat stress response

**Fig. 2:**
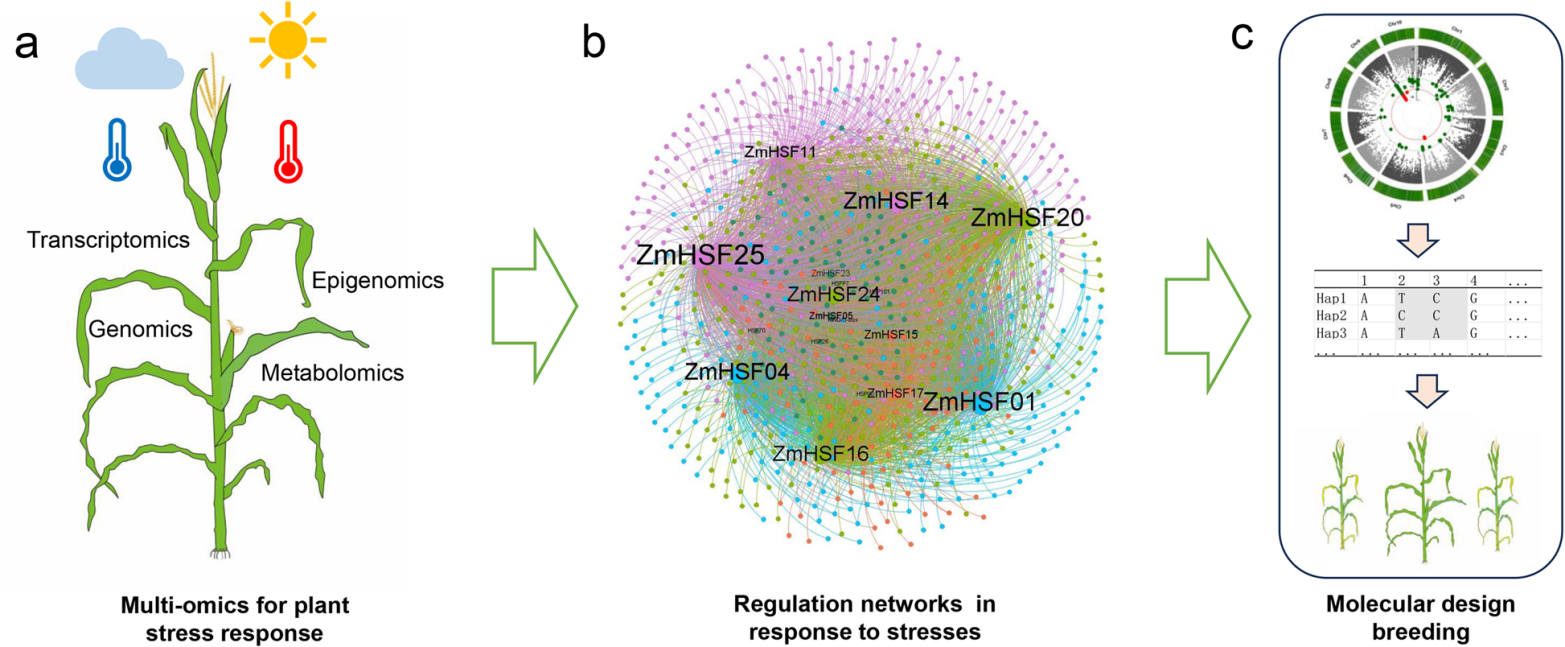
Systematic strategies to construct the regulatory network of heat stress response and enhance heat-tolerance maize breeding. **a** Multi-omics to study heat stress response of maize including genomics, transcriptomics, epigenomics and metabolomics. **b** The construction of regulatory network of *ZmHSFs* based on multi-omics data. **c** Modern genetic approaches to identify heat-tolerance quantitative trait locus or elite haplotypes for promoting heat-tolerance maize breeding

With the development of genome sequencing, multi-omics data helps to dissect multiple regulation pathways in different tissues under HT and heat stress (Derbyshire et al. 2022) (Fig. 2a). Compared to the regulation of leaves in response to HT, the mechanisms of pollen and silk in response to heat stress sense more important in relation to male sterility and crop yield (Chaturvedi et al. 2021; Khan et al. 2023). Single-cell multi-omics, including genomics, transcriptomics, epigenomics, and even proteomics, will provide unprecedented perspectives on the regulatory mechanisms of plant development and physiological responses (Yu et al. 2023). For example, the dynamic development atlas of maize pollen and meristem in response to HT would be interesting to be studied by using single-cell multi-omics to discover new cell types and new regulatory mechanisms acting during stress responses (Zhu et al. 2023). High throughput methods needed to be developed to profile the dynamic TFs and epigenetic binding landscape. For example, a low-cost and high-throughput *in vivo* chromatin profiling method tsCUT&Tag (a transient and simplified cleavage under targets and tagmentation) could have great potential for profiling the transcription factor binding landscape across plant development (Wu et al. 2021b). In addition, machine learning approaches and high-throughput phenomics coupled with precision gene-editing in crop species are likely to aid the breeding of stress-tolerant varieties to confront the daunting challenges of the incoming decades (Wu et al. 2021a). The integration of multi-omics with systems biology would be helpful to identify potential candidate genes for crop improvements under environmental stress conditions (Yang et al. 2021) (Fig. 2b).

### The combination study of heat with drought stresses

As global climate change, the combined stresses of high temperature and drought occurred more frequently and extensively, which make more threats to crop growth and productivity (Hu et al. 2023). Maize is a sensitive crop to both drought and heat stresses, particularly at the reproductive stages of development. Although major progress has been made in understanding the molecular regulation of drought and heat, respectively during past decades (EI-Sappah et al. 2022; Singh et al. 2023; Yang et al. 2023), the influence and molecular regulations of the combined stresses of high temperature and drought on summer maize production remains largely unknown. Therefore, the combination study of maize in response to heat with drought stresses should be a focus for cultivating elite maize verities with maximum tolerance against drought and heat stress in the future.

### Modern genetic approaches for heat-tolerant crop breeding

Modern genetic approaches, such as QTL-mapping, bulked segregant analysis (BSA), GWAS and genome editing, have facilitated gene cloning to develop maize verities with the highest heat tolerance (Benavente and Giménez 2021). The identification of heat-tolerance quantitative trait loci or natural variations by next-generation bulked segregant analysis for breeding 4.0 from wild and landrace species or other genetic population materials may provide effective ways to breed heat-tolerant crops (Wang et al. 2023) (Fig. 2c). In addition, gene editing technologies based on the clustered regularly interspaced short palindromic repeats (CRISPR)-associated protein (Cas) system have great potential to create much more variable sites and elite haplotypes for promoting heat tolerance by the manipulation of HSR genes (Zhu et al. 2023). Genetic variability and the effect of heat stress on crops in the field also need to be thoroughly investigated in order to provide useful strategies that can ensure future plants to successfully adapt to environmental temperature fluctuations caused by global climate change.

## Data Availability

Not applicable.

## Abbreviations

ABA: Abscisic acid
BSA: Bulked segregant analysis
ChIP-seq: Chromatin immunoprecipitation sequencing
CRISPR: Clustered regularly interspaced short palindromic repeats
ER: Endoplasmic Reticulum
GWAS: Genome-wide association study
HSR: Heat stress response
HSF: Heat shock factor
HSP: Heat shock protein
HT: High temperature
lncRNA: Long non-coding circular RNA
miRNA: MicroRNA
QTL: Quantitative trait locus
ROS: Reactive oxygen species
SNP: Single nucleotide polymorphism
ta-siRNA: Trans-acting small interfering RNA
